# Magnetic resonance imaging of the natural history of *in situ *mammary neoplasia in transgenic mice: a pilot study

**DOI:** 10.1186/bcr2357

**Published:** 2009-09-04

**Authors:** Sanaz A Jansen, Suzanne D Conzen, Xiaobing Fan, Erica J Markiewicz, Gillian M Newstead, Gregory S Karczmar

**Affiliations:** 1 Department of Radiology, University of Chicago, 5841 South Maryland Avenue, MC 2026, Chicago IL 60637, USA; 2 Department of Medicine & The Ben May Department for Cancer Research, University of Chicago, 5841 South Maryland Avenue, MC 2115, Chicago IL 60637, USA

## Abstract

**Introduction:**

Because of the small size of *in situ *mammary cancers in mouse models, high-resolution imaging techniques are required to effectively observe how lesions develop, grow and progress over time. The purpose of this study was to use magnetic resonance (MR) imaging to track *in vivo *the transition from *in situ *neoplasia to invasive cancer in a transgenic mouse model of human cancer.

**Methods:**

MR images of 12 female C3(1) SV40 Tag mice that develop mammary intraepithelial neoplasia (MIN) were obtained. MIN is believed to be similar to human ductal carcinoma *in situ *(DCIS) and is considered a precursor of invasive tumors. Images were serially obtained from 10-21 weeks of age at 2-3 week intervals. MIN lesions were identified based on their morphology on MR images. Lesions were followed over time and several lesion features were measured including volume, growth rate and morphology. For those MIN lesions that progressed to invasive cancer the progression time was measured.

**Results:**

Overall, 21 MIN lesions were initially detected at an average initial volume of 0.3 ± 0.2 mm^3 ^with an average growth rate of -0.15 ± 0.66 week^-1^. Even though all mice were inbred to express the SV40 Tag transgene in the mammary epithelium and expected to develop invasive carcinoma, the individual MIN lesions took vastly different progression paths: (i) 9 lesions progressed to invasive tumors with an average progression time of 4.6 ± 1.9 weeks; (ii) 2 lesions regressed, i.e., were not detected on future images; and (iii) 5 were stable for over 8 weeks, and were demonstrated by a statistical model to represent indolent disease.

**Conclusions:**

To our knowledge, the results reported here are the first measurements of the timescale and characteristics of progression from *in situ *neoplasia to invasive carcinoma and provide image-based evidence that DCIS may be a non-obligate precursor lesion with highly variable outcomes. In addition, this study represents a first step towards developing methods of image acquisition for identifying radiological characteristics that might predict which *in situ *neoplasias will become invasive cancers and which are unlikely to progress.

## Introduction

The processes that characterize and trigger progression of preinvasive ductal carcinoma *in situ *(DCIS) to invasive breast cancer remain elusive. DCIS is a heterogeneous disease in which neoplastic cells are confined by the basement membrane of lobuloductal or ductal lumen. Progression to invasive ductal carcinoma (IDC) is thought to occur by first degradation of the basement membrane, microinvasion of cancer cells into the surrounding stroma and growth of a solid tumor. The use of screening mammography has increased rates of detection of DCIS [1], which has in turn expanded knowledge about the biology of these earliest stage breast cancers. However, clinical imaging provides only a snapshot of tumor biology. Basic characteristics of DCIS development over time (i.e., growth rates and changes in morphology) and progression to IDC are still largely unknown [2].

Fundamental questions about the natural history of DCIS have remained unanswered largely because they are difficult to study in women. Due to obligate surgical excision of newly diagnosed cancers, subsequent lesion progression cannot be followed. A few studies have examined the outcome in a small number of women whose DCIS was initially misdiagnosed as benign disease, that is, treated by biopsy alone [3,4]. In one such study, 6 of 13 cases of DCIS progressed to invasive breast cancer in an average of nine years. In another, 11 of 28 women with misdiagnosed low-grade DCIS developed invasive carcinoma in the same quadrant, the majority within 15 years. These studies and others [5] have prompted some to suggest that DCIS may be over-diagnosed and over-treated [6-8] because not all will progress to invasive cancer. If this is the case, it is clinically important to identify predictive markers that can distinguish those DCIS that will remain indolent from those that will progress to life-threatening disease. Some studies suggest that a higher nuclear grade is related to an aggressive phenotype, because these lesions are more likely to recur as invasive tumors [9]. Although human studies provide important insights into the natural history of DCIS, they usually suffer from having small patient numbers, having a biased lesion population (i.e., only those DCIS that were initially misdiagnosed), performing interventions that could alter disease state and progression (i.e., biopsy or lumpectomy), and focusing on outcome rather than detailed measurements of lesion morphology or biology. It is difficult to fully understand DCIS development or the key steps involved in progression of *in situ *disease without detailed empirical data directly following DCIS as it develops and progresses over time.

Transgenic mouse models of human breast cancer provide an experimental framework with which to begin to understand the natural history of DCIS. Because of the small size of *in situ *mammary neoplasias in mouse models, high-resolution imaging techniques are required to effectively observe how lesions develop, grow and progress over time. Recently, our laboratory reported high-resolution *in vivo *magnetic resonance (MR) images of pre-invasive mammary intraepithelial neoplasias (MIN) in the simian virus 40 large T antigen (SV40 Tag) mouse model of human breast cancer [10]. This work demonstrated that both MIN and early invasive cancers could be identified and accurately classified based on their appearance on MR imaging, using histological analysis of the same lesions for verification. In the current study, we used these new MR techniques to follow *in situ *mammary neoplasias in SV40 Tag mice over time in individual animals. Specifically, the timescales and characteristics of the development and progression of *in situ *to invasive carcinoma were evaluated, and predictive markers of invasive progression were explored.

## Materials and methods

### Animals

All procedures were carried out in accordance with the University of Chicago's Animal Care and Use Committee approval. The C3(1) SV40 Tag transgenic mouse model of breast cancer was used. In this model, expression of SV40 large T antigen is targeted to the mammary gland in females via the C3 promoter. Female mice develop mammary neoplasias that resemble human intraductal neoplasias, including progression through atypical ductal hyperplasia (about eight weeks), MIN [11], which is similar to human DCIS (about 12 weeks), and invasive tumors (about 16 weeks) [12]. A total of 12 mice were selected for serial MR imaging. Four of 12 mice were selected for serial imaging every two weeks from ages 10 to 20 weeks. Eight of 12 were selected for serial imaging every three weeks from 12 to 21 weeks. An additional three 15-week-old C3(1) SV40 mice were selected for reproducibility studies of MR measurements.

### MR imaging experiments

The left inguinal mammary glands were selected for repeat *in vivo *imaging performed with a Bruker 9.4 Tesla magnet (Bruker-Biospin, Billerica, MA, USA). Axial gradient recalled echo (GRE) images with fat suppression (repetition time/echo time = 675/7 ms, field of view = 3.0 × 3.0 cm, matrix size = 256 × 256, number of excitation = 2, number of slices = 42, slice thickness = 0.5 mm, in-plane resolution = 117 microns and flip angle = 30°) across the entire sensitive volume of an open birdcage surface coil were obtained, so that images of the complete inguinal glands were acquired [10,13]. To facilitate spatial correlations between serial MR images, a fine polyethylene mesh about 3.0 cm × 2.0 cm in size with 3.0 mm spacing was embedded in partially deuterated agar and wrapped around each mouse. This grid produced a pattern on MR imaging that was used for registration of serial MR images so that lesions could be located and followed over time [10]. The grid was wrapped around the mouse during imaging, and using a Sharpie marker its position was marked on the skin of the mouse. These marks guided placement of the grid in a similar position upon repeat imaging. To assess the reproducibility of MR measurements, mice (n = 3) were reimaged under different conditions, including altered positioning in the coil, slice selections and imaging at different times. Animals were anesthetized prior to imaging experiments, and anesthesia was maintained during imaging at 1.5% isoflorane. The temperature, heart rate and respiration rate were monitored with data taken every minute, and the respiration rate was used to obtain gated images.

### Lesion identification

**Figure 1:**
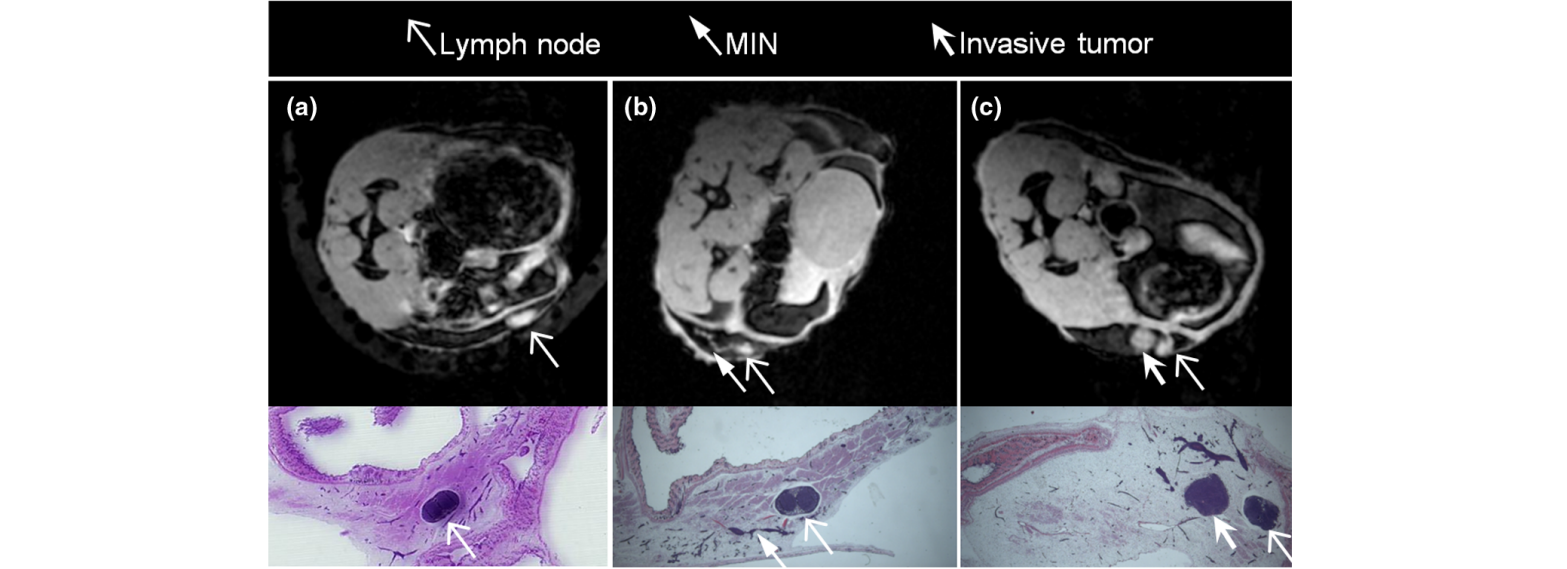
*In vivo *axial gradient recalled echo MR images and corresponding H&E-stained sections from a prior study [10]. The magnetic resonance (MR) images and H&E-stained sections represent different orientations, as each MR image represents only one cross-sectional slice through the mammary gland while the histologic sections show the entire gland. During imaging, the mammary glands are attached to the skin of the mouse, and are therefore wrapped around the body of the mouse. For excision, the glands are peeled back from the body of the mouse and laid flat, so that coronal H&E-stained sections can be obtained. We used an agar grid (a polyethylene mesh embedded in partially deuterated agar) to register the axial MR images with the H&E-stained sections. **(a) **Normal mammary gland, with intramammary lymph node, **(b) **lymph node and mammary intraepithelial neoplasia (MIN), **(c) **lymph node and small tumor. For each MR image, the display field of view is approximately 2.0 × 2.0 cm, and in-plane resolution is 117 microns.

In a prior study, we found that MR images acquired with a GRE pulse sequence demonstrated high sensitivity and specificity for both MIN and early invasive tumors (Figure 1) [10]. C3(1) SV40 Tag mice were imaged at various stages of cancer development and sacrificed afterwards to perform detailed correlations with histology using an agar grid. Several lesion features were evaluated, including morphology based on a simplified version of the Breast Imaging Reporting and Data System (BI-RADS) lexicon [14] as follows: type (mass or nonmass), shape/distribution (for mass lesions: round, oval, lobular or irregular; for nonmass lesions: linear, ductal or segmental), margins (for mass lesions only: smooth or irregular) and pattern (for mass lesions: homogeneous or heterogeneous; for nonmass lesions: homogeneous, stippled or clumped). We found that the type descriptors 'mass' and 'nonmass' were highly specific to invasive tumors and MIN, respectively. The results from our previous studies provided the basis for the present work by demonstrating that: (i) all MR findings in the gland correspond to MIN or invasive cancer, that is, there are no false positives; (ii) MIN, early invasive tumors and lymph nodes can be reliably identified based on morphology; and (iii) an agar grid can be used to localize and follow lesions over time. This work also demonstrated that image-based assessments of disease stage (i.e., *in situ *vs. invasive) are highly suggestive of true histology, but do not constitute definitive evidence thereof.

### Analysis of lesion features and development

**Figure 2:**
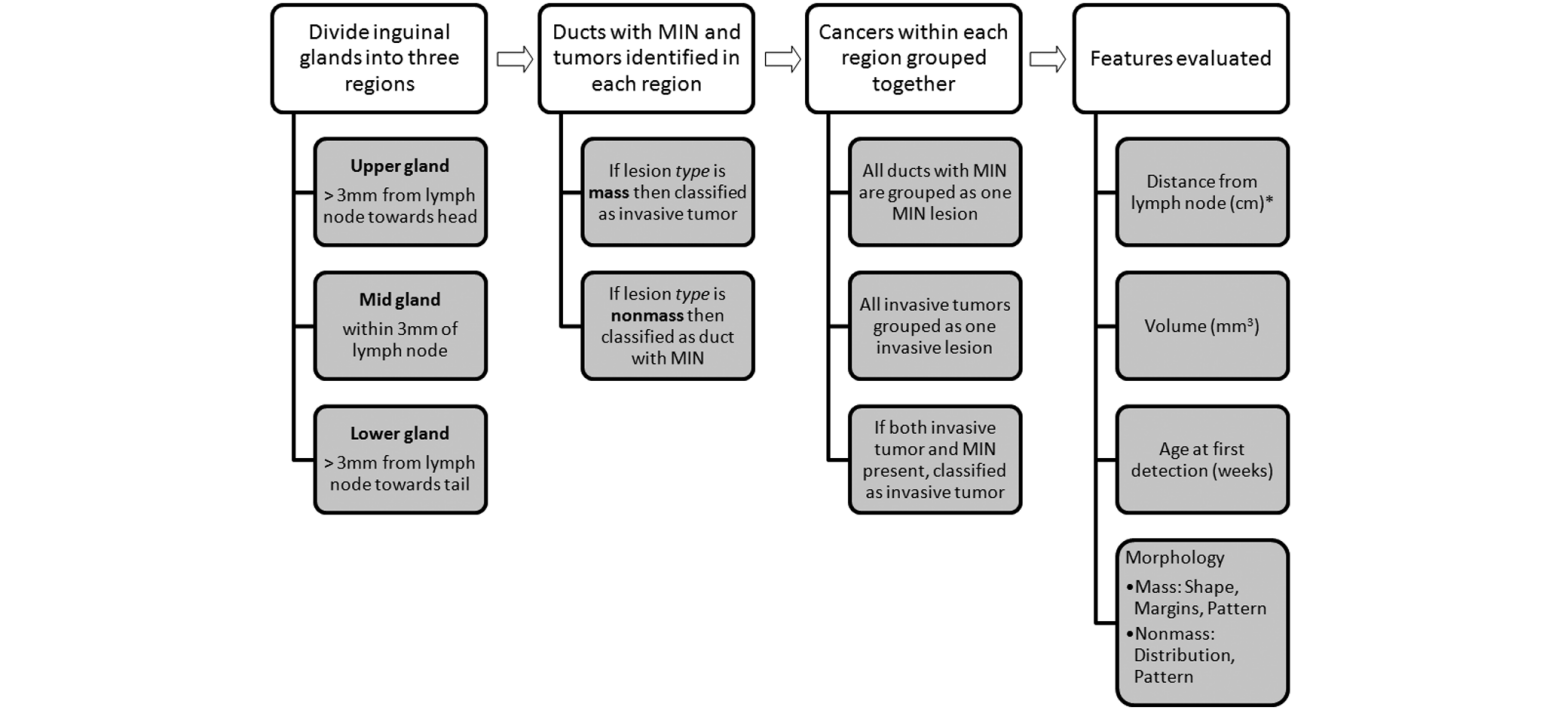
Flowchart demonstrating method for analyzing inguinal mammary glands for early murine mammary cancer. MIN = mammary intraepithelial neoplasia.

All image analysis was performed using software written in Interactive Data Language (IDL) (Research Systems, Inc., Boulder, CO, USA). The images of mouse mammary glands were analyzed in a manner analogous to methods used when evaluating human breast images. In women, cancers are often assigned a location by dividing the breast in quadrants relative to the nipple: upper-outer, upper-inner, lower-outer and lower-inner. Cancers within a quadrant are usually grouped as one, and the worst pathology determines the overall diagnosis; in other words, an invasive cancer with nearby extensive DCIS is considered an invasive tumor. For the mice, we proceeded with a similar analysis (Figure 2). The inguinal mammary glands were divided into three regions, this time using the intramammary lymph node as a reference point. Regions were examined to identify all ducts with MIN and invasive tumors, using the morphologic classification of lesion *type *(as defined above). Lesions within each region were grouped together (if necessary) and the following features were then evaluated: age at initial lesion detection (weeks), volume (mm^3^), further morphologic classification (as above, shape/distribution, margins and pattern) and distance from the intramammary lymph node (mm).

Using the agar grid and lesion location relative to the intramammary lymph node, lesion development was followed over time. This could only be assessed in cases where the lesion had been imaged at least twice. Growth rate was calculated according to the following equation:

- where *V *is lesion volume (mm^3^), *t *is the time in weeks, and α is the growth rate (per week). This was calculated separately for MIN (α_MIN_) and invasive tumors (α_tumor_). Changes in morphology as lesions developed were also examined separately for *in situ *and invasive tumors.

Reproducibility of MR assessments of lesions type (as 'nonmass' or 'mass'), morphology descriptors and size was evaluated for three mice with imaging repeated under different conditions.

### Analysis of MIN progression into invasive cancer

Each MIN was followed over time to determine whether invasive cancer developed in that region in the future. Specifically, if any invasive tumor developed in the same region on subsequent images, the lesion was classified as having progressed and the progression time T_prog _(in weeks) was calculated: T_prog _= age at initial detection of invasive tumor - age at initial detection of MIN. The average progression time μ_prog _and the standard deviation of progression times σ_prog _were then calculated. If an invasive tumor was not found in subsequent images, the latency time T_lat _(in weeks) was determined: T_lat _= age at final imaging session - age at initial detection of MIN. To determine whether some latent lesions truly represented non-progressing or 'indolent' disease, a threshold T_0 _was found so that MIN lesions with T_lat _> T_0 _could be considered a biologically distinct class of 'indolent' lesion. Details of the statistical method used are in the following section. In addition, the prior images of all invasive tumors were evaluated to determine how many were preceded by MIN.

We tested if a larger volume, earlier age at initial detection and a larger growth rate (α_MIN_) were predictors of whether that MIN lesion would progress in the future to an invasive tumor. In addition, to test whether cancer progression is related to lesion location in the gland, the distance from the lymph node was also evaluated as a predictor. The average value of each of these parameters was calculated separately in both progressing and indolent MIN, and compared using the Student's *t*-test. Receiver operating curve (ROC) analysis (ROCKIT 0.9B Beta Version, Charles E. Metz, University of Chicago, IL, USA) was performed to determine the diagnostic accuracy for each parameter in the task of distinguishing progression from indolent MIN. For each parameter, ROC analysis yielded area under the curve (A_z_) values that quantified diagnostic accuracy.

### Statistical method for identifying indolent MIN

We consider two groups of MIN: (i) lesions in which progression to invasive cancer was observed, with average time to progression T_prog _= μ_prog _± σ_prog_; and (ii) lesions in which progression to invasive cancer was not observed, with a range of latency times T_lat_. Our goal is to determine which lesions in the second group, if any, truly represent non-progressing or indolent disease. We begin first with the population of MIN lesions in which progression to invasive cancer was observed. We assume the progression time T_prog _for each lesion is normally distributed N(μ_prog_, **σ**_prog_), and denote N_prog _as the number of lesions in the progressing group. We next consider a subset of the latent MIN lesions with latency times T_lat _longer than a threshold T_0_, and denote N_0 _the number of lesions with T_lat _> T_0_. The probability that after randomly drawing *n *= N_prog_+ N_0 _lesions from N(μ_prog_, **σ**_prog_), we have selected at most N_prog _lesions with T_prog _< T_0 _can be found using cumulative form of the binomial distribution, given by the following density function:

- where *p *is the probability that one progressing lesion can have T_prog _< T_0 _(can be calculated using the cumulative distribution function of the normal distribution) and *k *= N_prog_. Thus, for each T_0 _we obtain a probability that lesions with T_lat _> T_0 _are part of the progressing group.

## Results

### Lesion features and development

Overall, 2 of 12 mice did not develop any cancerous lesion in the imaged inguinal glands. In the remaining 10 mice, a total of 21 MIN lesions developed. In eight of these mice a total of 14 invasive tumors developed (not always preceded by MIN in the same region). The lesion features at initial detection are summarized in Table 1. Most MIN lesions developed in the mid (10/21) or lower (9/21) gland regions, at a mean (± standard deviation) age of 12.7 ± 2.6 weeks and an average initial volume of 0.34 ± 0.22 mm^3^. MIN typically presented in a ductal or segmental shape and a homogenous pattern. Invasive tumors also developed predominantly in the mid (8/14) or lower (5/14) gland region. Tumors appeared at an average age of 16.3 ± 3.2 weeks at an initial volume at the time of detection of 17.2 ± 41.6 mm^3^. The latter value was skewed due to two tumors that presented initially at a very large volume (>120 mm^3^); excluding these two very large tumors reduced the average initial tumor volume to 1.96 ± 2.01 mm^3^. Both of the very rapidly growing large tumors were observed to have developed only three weeks after the *in situ *phase in the same animal. The typical invasive tumor morphology at initial detection was a round shape with smooth margins and a homogeneous pattern.

**Table 1:** The features at initial detection of MIN and early invasive cancers

Feature at initial detection		MIN (n = 21)	Invasive cancers (n = 14)
**Region**	Upper gland	2 (9%)	1 (7%)
	Mid gland	10 (48%)	8 (57%)
	Lower gland	9 (43%)	5 (36%)
**Distance from** **lymph node (mm)**		3.5 ± 1.8	6.6 ± 7.6
**Age (weeks)**		12.7 ± 2.6	16.3 ± 3.2
**Volume (mm^3^)**		0.34 ± 0.22	17.2 ± 41.6*
**Morphology**			
Type**	Nonmass	21 (100%)	0 (0%)
	Mass	0 (0%)	14 (100%)
**Distribution (Nonmass)**	Ductal	11 (52%)	--
	Segmental	9 (43%)	--
	Linear	1 (5%)	--
**Pattern (Nonmass)**	Clumped	2 (10%)	--
	Homogeneous	14 (67%)	--
	Stippled	5 (23%)	--
**Shape (Mass)**	Irregular	--	3 (21%)
	Round	--	9 (64%)
	Lobular	--	2 (14%)
**Margins (Mass)**	Smooth	--	10 (71%)
	Irregular	--	4 (29%)
**Pattern (Mass)**	Heterogeneous	--	4 (29%)
	Homogeneous	--	10 (71%)

*If we exclude two tumors that initially presented at over 100 mm^3^, the average was 1.96 ± 2.01 mm^3^.

** Please note that by definition 'mass' lesions were classified as invasive tumors, while 'nonmass' lesions were classified as mammary intraepithelial neoplasia (MIN).

**Figure 3:**
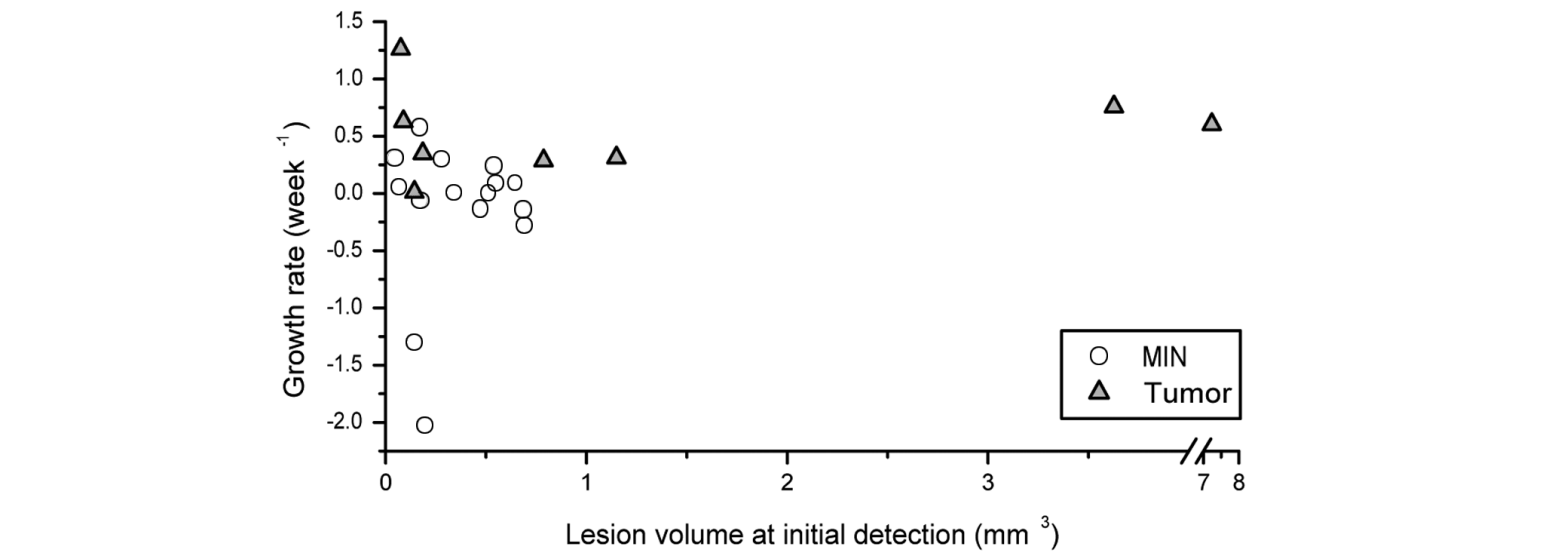
Scatter plot of growth rates and lesion volume at initial detection in nine mice: 15 MIN lesions (in nine mice) and eight invasive tumors (in seven mice). Note that this plot displays only those lesions in which a growth rate could be calculated, i.e., that were imaged at least twice as mammary intraepithelial neoplasia (MIN) or invasive tumor. Six of the MIN and invasive tumor data points (in six mice) represent growth rates of progressing MIN lesions and the invasive phase, subsequently found in the same region of the mammary gland.

**Figure 4:**
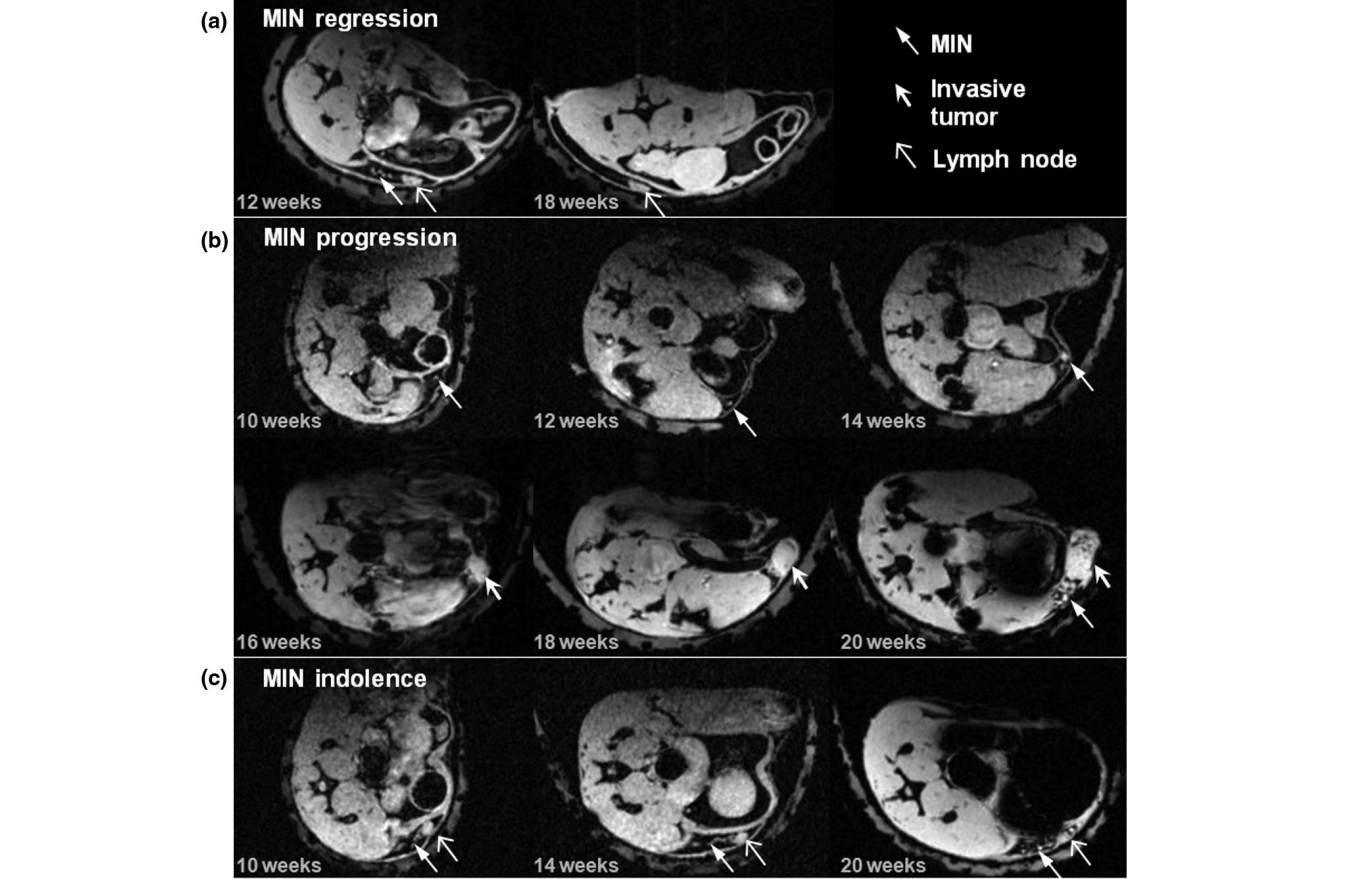
**Examples of MIN progression in three different mice**. **(a) **An example of possible mammary intraepithelial neoplasia (MIN) regression. MIN is visible at 12 weeks of age (left) beside lymph node, but cannot be found six weeks later anywhere near the lymph node. Here, only one slice is shown demonstrating absence of a lesion near the lymph node (right). **(b) **MIN is first detected early on at 10 weeks of age, and in this axial MR image appears in cross section. The duct grew more distended at 12 and 14 weeks, and by 16 weeks had become an invasive tumor. The tumor then continued to grow and by 20 weeks was quite large. Interestingly, at 20 weeks it appeared that new MIN had developed close to the tumor. **(c) **MIN has developed at 10 weeks and does not progress to invasive cancer. Field of view for all images is 3.0 × 2.0 cm, and in-plane resolution is 117 microns.

The subsequent development of MIN was studied in those 15 of 21 lesions in nine mice that were detected and classified as MIN at least twice by MRI. Interestingly, the average growth rate was slightly negative α_MIN _= -0.15 ± 0.66 weeks. Several lesions exhibited close to zero growth (Figure 3); two in particular exhibited considerably negative growth because on subsequent imaging they were no longer detected (Figure 4a). This could be evidence of *in situ *cancer disappearance; at the least, it indicates the lesion has substantially reduced in size, that is, it had regressed. Some of the MIN lesions also exhibited morphological changes as they developed. Two of 15 lesions exhibited a change in lesion shape from ductal to segmental. More significantly, 8 of 15 lesions showed changes in lesion pattern from homogeneous to clumped or stippled.

Early invasive tumors exhibited less variability compared with MIN as they grew over time. For the 8 of 14 invasive tumors that were imaged at least twice, the average growth rate was 0.53 ± 0.38 weeks, significantly higher than that of MIN (*P *= 0.003). Although the growth rates of invasive tumors varied considerably (Figure 3), none of the invasive cancers reduced in size over time. Furthermore, tumors maintained similar morphologic characteristics as they developed. Only two of eight tumors changed morphology over time: one transitioned from a round to lobular shape, and the other from a round mass with smooth margins to an irregular mass with irregular margins. This suggests that the growth patterns of early invasive tumors are more stable than MIN lesions.

In preliminary reproducibility experiments, we found that the classification of lesion type as 'mass' or 'nonmass' is robust and does not change with different setups. In addition, measurements of early invasive tumor morphology and size were reproducible. For 'nonmass' lesions, however, morphologic descriptors of distribution and pattern were not consistent on repeat imaging.

### Progression of MIN into early invasive cancers

With the caveat that image-based assessments of lesion histology (i.e., *in situ *vs. invasive) are highly predictive but not conclusive proof of true disease stage, we proceeded to use image-based features to analyze *in situ *neoplastic progression. Based on image-based evidence alone, nine of 21 MIN lesions progressed into invasive cancers with an average progression time of T_prog _= 4.6 ± 1.9 weeks (Figure 4b). Of these, five MIN lesions progressed to invasive cancer in two to three weeks, three within six to seven weeks, and one in eight to ten weeks. Eleven of 21 MIN did not progress to invasive tumors with an average latency time of T_lat _= 5.8 ± 3.8 weeks (Figure 4c). Of these, four did not progress for at least two to three weeks, two for at least six to seven weeks and five were stable for at least eight to ten weeks (i.e., T_lat _≥ 8 weeks). One of 21 MIN developed at the last imaging time point (21 weeks of age) and thus subsequent development was unknown. Nine of 14 invasive tumors were preceded by MIN that was detected by MR imaging; MIN was not detected in the prior images of four tumors, and one tumor was detected at the first imaging session (age 12 weeks) and thus prior history was unknown.

We compared the growth rates of those MIN lesions that progressed to invasive cancer to the growth rates of the invasive tumors that subsequently developed in the same region. This was possible in six of the nine MIN lesions that progressed to invasive cancer. There was a tendency for increased MIN growth rate to be correlated with an increased growth rate of the subsequent invasive cancer; for the three MIN lesions with the lowest growth rates (average α_MIN _= -0.06 ± 0.07 per week) the invasive tumors that developed in the same region had an average invasive growth rate of α_tumor _= 0.36 ± 0.38 per week. In comparison, for the three MIN lesions with the highest growth rate (average α_MIN _= 0.31 ± 0.25 per week) the invasive tumors that developed in the same region had a higher average growth rate of α_tumor _= 0.83 ± 0.37 per week, although this difference was not significant (*P *= 0.19).

Using the statistical method outlined in the Methods, and with N_prog _= 9, μ_prog _= 4.56 and σ_prog _= 1.9 weeks, we found that there was a less than 0.5% probability that lesions with T_lat _greater than or equal to eight weeks could be from the same population as progressing MIN. In other words, according to our methods, MIN lesions with T_lat _of eight weeks or more represented indolent disease that is biologically different from the MIN lesions that progress.

There was a trend for indolent MIN lesions to develop earlier than lesions that went on to progress to invasive cancer, to be closer to the lymph node and to have a lower growth rate compared with progressive MIN (Table 2). However, due to the small sample size, the confidence intervals of the A_z _values were large.

**Table 2:** Values of lesion features in progressing vs. indolent MIN

Feature	Progressing MIN (n = 9)	Indolent MIN (n = 5)	*P *value	**A**_**z**_
Age at first detection (weeks)	12.3 ± 2.3	10.6 ± 0.9	0.07	0.83 (0.51 to 0.97)
Maximum volume (mm^3^)	0.55 ± 0.66	0.67 ± 0.87	0.81	0.48 (0.19 to 0.78)
Growth rate (per week)**	0.21 ± 0.29	-0.66 ± 0.952	0.11	0.83 (0.45 to 0.98)
Distance from lymph node (mm)	4.2 ± 2.2	2.7 ± 1.6	0.18	0.75 (0.43 to 0.94)

** Student t-test comparisons yielded *P *values shown above. For the area under the curve (A_z_) values, numbers in parentheses represent 95% confidence intervals.

Growth rate could only be measured for those lesions that were imaged as mammary intraepithelial neoplasia (MIN) at least twice.

## Discussion

We have used serial MR imaging to study the natural history of *in situ *neoplasia in a transgenic model of human breast cancer. The timescales of neoplastic initiation and progression to invasive cancer in C3(1) SV40 Tag mice that can only be derived from repeated non-invasive imaging were measured. Significantly, we found that even in these mice that are genetically predisposed to develop invasive carcinoma, a substantial proportion of *in situ *cancers did not progress to invasive tumors within 21 weeks of monitoring. To our knowledge, these results provide the first detailed, high-resolution measurements of early mammary cancer natural history in mice. Our work complements work by Abbey and colleagues that investigated malignant transformation of *in situ *mammary cancer in a transplantable tissue model, using positron emission tomography (PET) to provide metabolic characterization at lower spatial resolution (5 mm^3 ^voxel size compared with 0.0068 mm^3 ^in our study) [15,16].

In the present study, we measured changes in image-based features that are highly predictive of disease stage. As this was a serial imaging study, we could not perform histologic confirmation of the image-based findings. This underscores a significant challenge with noninvasive imaging of disease progression: it is very difficult to determine lesion histology without sacrificing the animal, thereby losing longitudinal information. With larger and more detailed sensitivity/specificity studies that correlate image-based features with a wide variety of histologic presentations, this limitation can be mitigated.

If image-based measurements are to be robust predictors of disease stage, their reproducibility must be established. Our preliminary work suggests that assessment of *in situ *vs. invasive disease (via determination of lesion type) is reproducible, as are measurements related to size and morphology of early invasive tumors. However, *in situ *lesion morphology (i.e., distribution or pattern) may not be adequately reproducible. Further studies are needed to quantify reproducibility of MR measurements of early murine mammary cancer.

The C3(1) SV40 Tag mouse model is being used for a wide variety of studies, ranging from evaluating effects of interventional and preventive therapies [17-30], to understanding molecular and genetic alterations occurring at various stages of disease progression [31-36]. Our results contribute new observations regarding this mouse model of breast cancer. MIN lesions grow slowly on average, and can both progress to invasive tumors or remain indolent, as has been suggested to be true for DCIS in women [2-4]. This is consistent with additional stochastic mutations taking place as secondary transformation events beyond expression of Tag as required for cancer progression in this model [33]. We have also found evidence for *in situ *cancer regression, which if validated in larger numbers with detailed pathology correlation, would be direct demonstration of spontaneous breast cancer regression [37-39]. The heterogeneity of progression paths demonstrates that the C3(1) SV40 mouse model may be a good candidate for assessing the effect of therapies that delay the progression of DCIS. Early invasive tumors show less variability in morphology as they grow compared with MIN. Although there was a wide variability of growth rates of early invasive cancers, overall they grew much faster than *in situ *cancers, and none decreased in size or regressed. There was a trend for increased MIN growth rate to be a predictor of both the eventual development of invasive carcinoma, and a higher invasive tumor growth rate, in the same region. Unfortunately, in this pilot study the number of cases was too small to draw conclusions with statistical significance.

It is important to note that our results pertain to this specific mouse model; it will be important to establish imaging techniques and assess similar characteristics in other mouse models of human breast cancer. If some features can be found that persist across mouse models, they may ultimately demonstrate applicability to human disease.

The natural history of breast cancer is still an open question, and there are many theories of the mechanisms governing the growth and progression of early breast cancers in women. The 'angiogenic switch' is thought to be a crucial step during breast tumorigenesis, and has been hypothesized to occur at or before the *in situ *stage [40]. Franks and colleagues have used non-linear mathematical models to predict that invasion will occur at the middle of ducts distended by DCIS due to increased mechanical pressure [41-43]. Tabar and colleagues suggest that true *in situ *lesions in fact originate in lobules, and that a separate more aggressive disease representing a duct-forming invasive carcinoma is being incorrectly included with other *in situ *cancers [44,45]. Due to an absence of empirical data of the detailed morphologic changes and other changes that occur during progression of *in situ *cancers, such theories may be difficult to evaluate. Our work and extensions thereof, for example the use of dynamic contrast-enhanced MR imaging to probe changes in vasculature, can provide detailed and direct measurements of tumorigenesis on which these mathematical and physiologic models of disease initiation and progression can be evaluated.

There are several limitations to this study. First, lesion morphology was assessed using 2D axial slices rather than a 3D rendering of the inguinal glands. This could compromise the assessment of lesion morphology, particularly of MIN located in the lower gland area.

Second, there may have been some errors in lesion identification. Although the descriptors of 'nonmass' and 'mass' are highly specific to MIN and invasive tumors, respectively, the 'mass' descriptor is not perfectly correlated [10] implying that some MIN lesions may have been misidentified as invasive tumors. More generally, in this study it may have been difficult to distinguish focal MIN from invasive cancer, or to pinpoint the exact point of transition from MIN to invasion due to the two to three-week sampling interval. On the other end of the progression spectrum, distinguishing MIN from benign conditions, such as atypical ductal hyperplasia or epithelial proliferative diseases, may be a challenging task in mice as it is in women. A much larger sensitivity/specificity study will be required to better correlate a wider variety of image-based features with histology, in order to minimize such confusion so that disease stage can be assessed with increased confidence. Similarly, MR imaging of nontransgenic normal mice should be performed to document the presentation of normal murine mammary gland anatomy, upon which findings in transgenic models can be compared.

Third, the numbers of lesions studied was rather small, limiting the statistical significance of our findings. To address this, both an increased number of mice should be imaged as well as an increased number of mammary glands in each mouse (rather than only the inguinal glands on one side). In this way, cancer development can be assessed in the whole mouse, and data can be analyzed to determine whether lesions in the same animal can be considered as independent.

Fourth, in this study we did not consistently perform end-point histologic evaluation of imaged mammary glands.

Fifth, mice were followed to 20 or 21 weeks of age rather than until natural death, so that studies of cancer progression could not be performed past this age. Although the range of cancer development in this model spans 10 to 24 weeks of age, and most female mice must be sacrificed at this time due to increased tumor burden in the mammary glands, additional time points should be acquired to definitively assess *in situ *cancer progression.

Sixth, changes in the parenchyma that preceded the development of MIN could not be easily observed because of the poor signal-to-noise ratio of the normal tissue. Recent improvements in imaging methods have provided greatly enhanced images of normal parenchyma, opening up the possibility of studying changes in the normal mammary glandular tissue that are precursors to cancer development.

Finally, the new framework we have presented for analyzing early carcinogenesis in mice may need improvement. For example, the number of MIN lesions that progressed to invasive tumors may have been over-estimated. Our criterion was only that an invasive tumor appeared in the same region on subsequent imaging; however, this tumor may have been independent of the original MIN detected previously. The transition from MIN to invasive tumors was rarely observed directly. In addition, cancer growth rates could only be calculated for lesions imaged at least twice, that is, 15 of 21 MIN and 8 of 14 invasive tumors. The remaining lesions were excluded from any analysis of growth rates, which may have introduced a bias. These two limitations could be addressed by conducting serial imaging at higher frequency (i.e., every few days) so that each MIN lesion can be definitively linked with its subsequent invasive phase, and so that growth rates can be measured for all. Lastly, the statistical model we used to identify indolent lesions could most likely be improved or modified.

In prior work, we introduced MR imaging methods for imaging early murine mammary neoplasias and invasive cancers, and subsequently reported on how those techniques could be used to better interpret clinical MR imaging of the breast [46]. Here, we have established a new role for MR imaging in preclinical studies of the natural history of early breast cancer. In future work, we plan on performing more detailed studies of carcinogenesis, by imaging more frequently and at higher resolution. In addition, we will explore additional MR imaging techniques, such as dynamic contrast enhanced MR imaging, diffusion weighted imaging and high spectral-spatial resolution imaging, to probe the changes in vasculature and cellularity that occur during progression to invasive cancer. Finally, molecular imaging and gene/protein expression studies will be explored in conjunction with MRI to interrogate the molecular mechanisms involved in cancer initiation and progression.

## Conclusions

We have used longitudinal noninvasive imaging to gain new insights into the natural history of early mammary cancer in the C3(1) SV40 Tag mouse model of human breast cancer. We found image-based evidence that some *in situ *mammary cancers did not progress to invasive cancers, and investigated potential predictive markers of progression. This pilot study represents a first step towards detailed studies of functional and morphologic characteristics of mammary tumorigenesis, and developing methods for image acquisition and analysis that can predict which *in situ *cancers will become invasive and which would not. Such investigations would have an important impact on clinical management of patients with DCIS.

## Abbreviations

Az: area under the curve; BI-RADS: Breast Imaging Reporting and Data System; DCIS: Ductal carcinoma *in situ*; GRE: gradient recalled echo; IDC: invasive ductal carcinoma; IDL: Interactive Data Language; MIN: mammary intraepithelial neoplasia; MR: magnetic resonance; PET: positron emission tomography; ROC: receiver operating curve; SV40 Tag: simian virus 40 large T antigen.

## Competing interests

The authors declare that they have no competing interests.

## Authors' contributions

SJ conceived of the study and experiment design, conducted the imaging experiments, performed the data analysis and drafted the manuscript. SC participated in conception and design of the study and provided transgenic mice for imaging. XF helped to draft the manuscript and perform data analysis. EJ is the veterinary technician that participated in all imaging experiments. GM helped conceive and design the study. GK conceived of the study and experiment design, and participated in its coordination and helped to draft the manuscript. All authors read and approved the final manuscript.
